# The association between triglyceride-glucose index, atherogenic index of plasma, systemic immune-inflammation index, and mortality in patients with acute coronary syndrome: the direct effects of glucose-lipid metabolism and U-shaped immune modulation in mortality risk

**DOI:** 10.3389/fcvm.2025.1604284

**Published:** 2025-07-25

**Authors:** Weichen Luo, Zaixiao Tao, Xinxin Li, Yang Xu, Chun Yang, Rui Sun, Mi Wang, Zhenjun Ji, Genshan Ma

**Affiliations:** Department of Cardiology, Zhongda Hospital, School of Medicine, Southeast University, Nanjing, Jiangsu, China

**Keywords:** glucolipid metabolism, inflammation, nonlinear relationship, acute coronary syndrome, triglyceride-glucose index, atherogenic index of plasma, systemic immune inflammation index

## Abstract

**Background:**

Cardiovascular disease (CVD) remains the leading global cause of death, with inflammation and glycolipid dysregulation as key drivers of atherosclerosis progression. While triglyceride-glucose index (TyG) and Atherogenic Index of Plasma (AIP) are linked to cardiovascular risk, their prognostic value in Acute Coronary Syndrome (ACS) patients, particularly Acute Myocardial Infarction (AMI) patients, and mediating role of systemic inflammation remain unclear. This study investigates the relationship between glycolipid metabolism, systemic inflammation, and mortality in ACS patients.

**Methods:**

In this single-center retrospective study, 3,861 ACS patients were analyzed. Glycolipid metabolism was assessed using the TyG and AIP index, while the systemic immune-inflammation index (SII) evaluated inflammatory status. Missing data were addressed with random forest multiple imputation. Statistical analyses included the Least Absolute Shrinkage and Selection Operator (LASSO) regression for variable selection, generalized linear modeling, restricted cubic splines (RCS) for nonlinear associations, and the Mantel test for correlations between glycolipid metabolism and inflammatory markers. Additionally, multivariable logistic regression, RCS models, and mediation analysis were used to assess associations and pathways.

**Results:**

Elevated TyG index linearly increased mortality risk in ACS patients (Odds Ratio (OR) = 1.64, 95% Confidence Interval (CI):1.07–2.52) and AMI subgroups (OR = 1.56, 95% CI:1.00–2.42), with minimal SII mediation (ACS:3.97%; AMI: non-significant).The AIP index directly increased mortality risk (ACS: Beta coefficient (β) = 0.076; AMI: β = 0.091, *p* < 0.001), partially offset by SII's negative mediation (ACS:−6.6%; AMI:−7.8%). SII showed U-shaped mortality associations in ACS and AMI patients, with the lowest risk around 450–900  ×  10⁹/L. Age ≥ 75 (ACS: OR = 8.35; AMI: OR = 10.12), STEMI diagnosis (ACS: OR = 1.46; AMI: OR = 1.53), and elevated total cholesterol (ACS: OR = 1.50; AMI: OR = 1.40) were independent mortality predictors. Increased HDL-C (ACS: OR = 0.198; AMI: OR = 0.280) was an independent protective factor.

**Conclusion:**

The TyG and AIP index independently predict mortality in ACS and AMI patients through direct metabolic toxicity rather than inflammatory mediation.SII exhibits a U-shaped mortality association, reflecting bidirectional immune regulation (tissue repair vs. damage), with an optimal threshold range of 450–900 × 10^9^/L to guide anti-inflammatory strategies. Findings support metabolic-inflammatory risk stratification, prioritizing glycolipid metabolic dysregulation intervention in acute events while dynamically monitoring SII to balance immune homeostasis.

**Trial registration:**

Approved by Zhongda Hospital Ethics Committee (2020ZDSYLL164-P01); retrospectively registered.

## Graphical Abstract

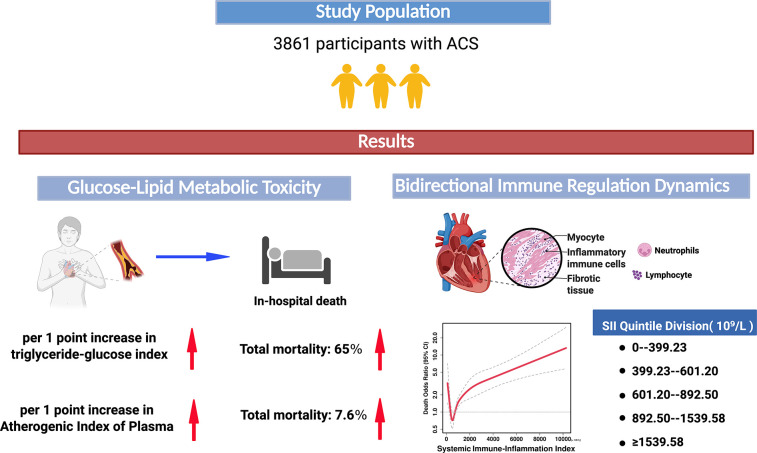

## 1 Background

Cardiovascular disease (CVD) is the foremost challenge in global public health, with persistently high mortality rates (1). Atherosclerosis serves as the pathological basis for atherosclerotic cardiovascular disease (ASCVD). Its occurrence and progression are jointly driven by disorders of glucose and lipid metabolism along with chronic inflammation. The inflammatory response is a key factor affecting prognosis throughout the disease (2, 3). The process begins with endothelial dysfunction and progresses to the formation of vulnerable plaques, characterized by lipid accumulation, inflammatory cell infiltration, and extracellular matrix remodeling. These changes ultimately lead to acute coronary syndrome (ACS) through platelet activation (4, 5). Inflammatory indices derived from complete blood count (CBC), such as the neutrophil-to-lymphocyte ratio (*N*LR) and systemic inflammation response index (SIRI), are closely related to cardiovascular event prognosis. For instance, the risk of major adverse cardiovascular events (MACE) increases by 1.153 times in acute myocardial infarction (AMI) patients with elevated SIRI (95% CI 1.251–3.705) (6, 7).

The role of glucose and lipid metabolism disorders in the chronic course of atherosclerosis begins in the prediabetic stage (8). Insulin resistance, a core marker of these metabolic disorders, significantly manifests early in the prediabetic stage and progresses concurrently with metabolic abnormalities (9). Hyperglycemia induces mitochondrial dysfunction and excessive generation of reactive oxygen species (ROS), activating inflammatory mediators and leading to oxidative stress and endothelial dysfunction (10, 11). At the same time, advanced glycation end products accelerate plaque progression by enhancing inflammatory signaling (12). Lipid metabolic dysregulation drives pathological progression via inflammatory mechanisms. Triglyceride-rich lipoprotein (TGRL) metabolic abnormalities generate atherogenic remnants. Macrophages internalize these remnants, transforming them into foam cells. Foam cell deposition in vascular walls triggers inflammatory cytokine secretion, disrupting collagen synthesis/degradation equilibrium and increasing plaque rupture susceptibility (13, 14). Furthermore, TGRL remnants have been demonstrated to induce endothelial cells to release pro-inflammatory cytokines and adhesion molecules, promoting leukocyte infiltration. The prothrombotic state is formed through ROS bursts and the activation of pro-apoptotic pathways, increasing the risk of acute cardiovascular events (15–17). The glucose and lipid metabolism disorders act synergistically through multiple inflammatory signaling pathways, ultimately leading to plaque destabilization and the occurrence of cardiovascular events (18).

In the context of metabolic assessment, traditional tools for evaluating insulin resistance, such as the homeostasis model assessment of insulin resistance (HOMA-IR), are limited by high detection costs and standardization challenges. In this context, the triglyceride-glucose index [TyG, ln(triglycerides × glucose/2)] has emerged as a preferred indicator due to its simplicity and sensitivity (19). In a comparative analysis, TyG has been shown to outperform HOMA-IR in predicting prediabetes, with a sensitivity of 84.0% (19). An increase of 1 unit in TyG has been associated with an 82% increase in the risk of major adverse cardiovascular events (MACE) following percutaneous coronary intervention (PCI) and a 157% increase in the risk of nonfatal myocardial infarction (20, 21). Furthermore, TyG has been shown to interact synergistically with inflammation. When it is combined with elevated high-sensitivity C-reactive protein (hs-CRP), cardiovascular risk increases by 1.3 times (HR = 1.300), with inflammation mediating 13.4% of TyG's effect and TyG mediating 7.9% of the inflammation effect (22). The plasma atherogenic index (AIP) is calculated as log10[triglycerides(TG)/High-Density Lipoprotein Cholesterol (HDL-C)], reflecting characteristics of atherogenic lipoproteins (23). AIP has been shown to have a significant positive association with the risk of cardiovascular events. The high AIP group exhibited a 56% increase in cardiovascular risk. In patients diagnosed with chronic coronary heart disease, an AIP ≥ 0.24 was associated with a significantly higher incidence of MACE (24, 25). Importantly, AIP also predicts MACE risk in type 2 diabetes patients, reflecting the comprehensive impact of glucose and lipid metabolism disorders on the cardiovascular system (26).

In summary, disorders of glycolipid metabolism drive atherosclerosis progression through inflammation. TyG and AIP, as sensitive markers of glycolipid metabolism, are optimized for risk stratification in association with inflammatory indicators. This study aims to address the gap in assessing the glucose and lipid metabolism and inflammation axis in ACS patients, particularly AMI. An observational cohort design will be employed to integrate TyG, AIP, and inflammation indices, exploring their prognostic value to provide evidence for precise prevention in high-risk populations of coronary heart disease, especially those with metabolic disorders related to AMI.

## 2 Methods

### 2.1 Study participants

This study is a single-center, retrospective, and observational research approved by the Ethics Committee of Zhongda Hospital, Southeast University (Ethics Approval Number: 2020ZDSYLL164-P01). A total of 3,861 patients were included in this study, comprising 1,140 patients with non-ST-segment elevation myocardial infarction (NSTEMI), 1,258 patients with ST-segment elevation myocardial infarction (STEMI), and 1,463 patients with unstable angina (UA) who were consecutively admitted to Zhongda Hospital between June 2013 and February 2018. The inclusion criteria were as follows: (1) Age > 18 years; (2) Diagnosed with acute coronary syndrome (ACS) according to the guidelines for the diagnosis and treatment of ACS. The exclusion criteria were as follows: (1) Presence of severe diseases (e.g., malignant tumors) with a life expectancy of less than six months; (2) Pregnant or breastfeeding women; (3) Presence of acute or chronic inflammatory diseases, such as chronic liver disease, chronic kidney disease, cardiogenic shock at admission, cardiac arrest (pre-hospital or in-hospital), autoimmune disorders, active infectious diseases, inflammatory bowel disease or other chronic inflammatory conditions.

### 2.2 Flowchart

**Figure 1:**
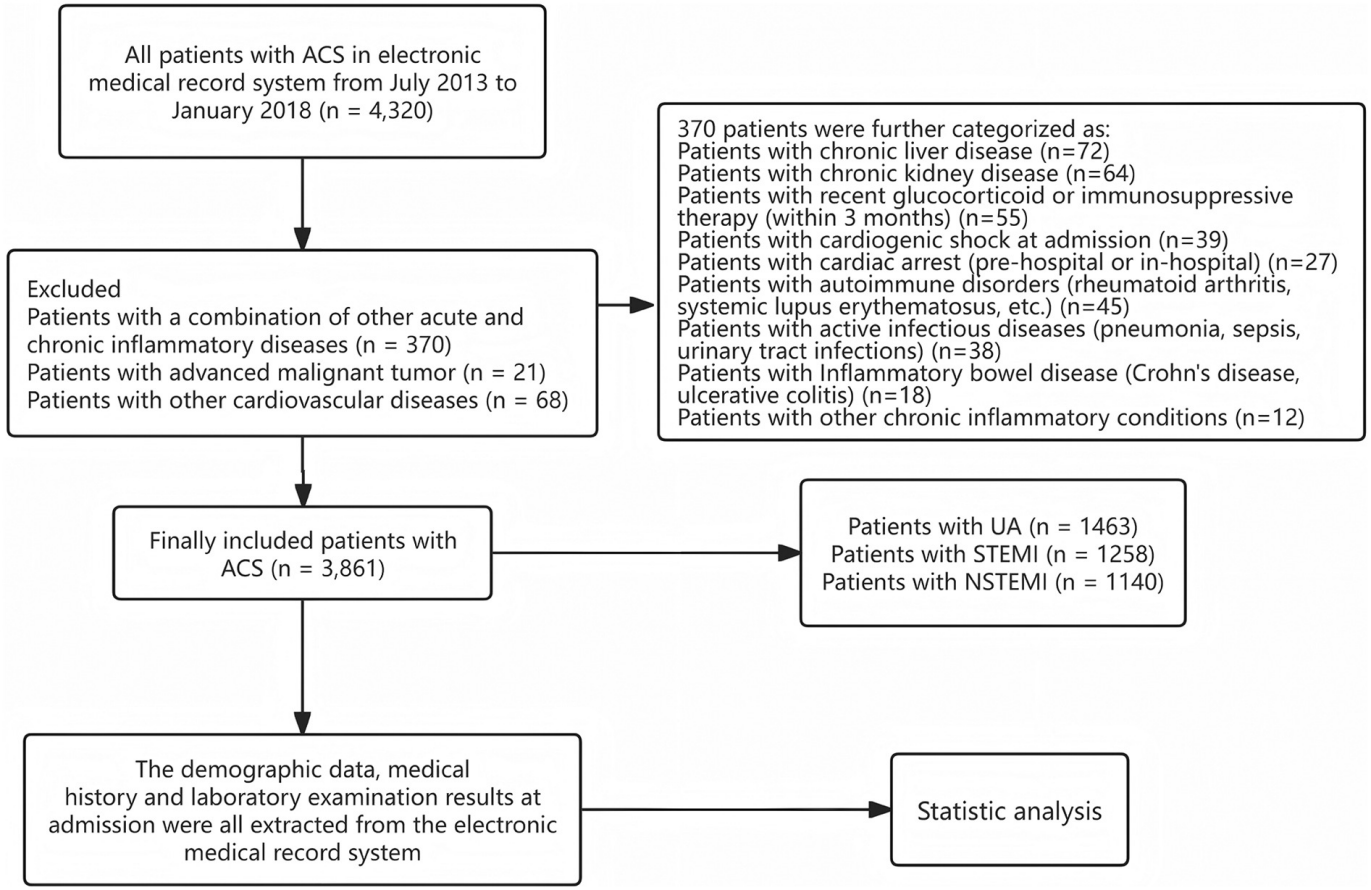
Flow chart of patients enrolling.

From July 2013 to January 2018, a total of 4,320 ACS patients were consecutively screened through the electronic medical record system. Among them, 370 patients with concurrent acute or chronic inflammatory diseases were excluded, as well as 68 patients diagnosed with other cardiovascular diseases. Additionally, 21 patients with malignant tumors were also excluded. Ultimately, this study included 3,861 ACS patients, consisting of unstable angina (UA), ST-segment elevation myocardial infarction (STEMI), and non-ST-segment elevation myocardial infarction (NSTEMI) (Figure 1).

### 2.3 Data collection and imputation

A comprehensive data collection process was implemented utilizing the medical record system (China Yidu Cloud System), encompassing patient demographic characteristics, medical history, laboratory, and imaging data. In-hospital death was defined as all-cause mortality due to cardiovascular or non-cardiovascular diseases, with all death events supported by medical records. Demographic data included age, sex, smoking history, and alcohol consumption history; medical history data included hypertension and diabetes history. The first laboratory test results after admission were extracted, including complete blood count, liver and kidney function biochemical tests, and coagulation function tests. Fasting blood glucose and lipid levels of all participants were assessed upon admission. Based on CBC data, the following inflammatory Indices were calculated: NLR, derived Neutrophil-to-Lymphocyte Ratio(dNLR), Monocyte-to-Lymphocyte Ratio(MLR), Neutrophil-to-Monocyte-to-Lymphocyte Ratio(NMLR), SIRI, and Systemic Immune-Inflammation Index(SII). The specific formulas are as follows: NLR = neutrophil count/lymphocyte count; dNLR = neutrophil count/(white blood cell count—lymphocyte count); MLR = monocyte count/lymphocyte count; NMLR = (monocyte count + neutrophil count)/lymphocyte count; SIRI = neutrophil count × monocyte count/lymphocyte count; SII = platelet (PLT) count × neutrophil (Neut) count/lymphocyte (Lymph) count (27). Additionally, the TyG index was calculated using the formula: ln [TG (mg/dl) × glucose(GLU) (mg/dl)/2]. The calculation formula for AIP is: log10 [TG (mg/dl)/HDL-C (mg/dl)]. The calculation formula for SII is: PLT (10⁹/L) × Nero (10⁹/L)/Lymph (10⁹/L). Missing data filling was done by R 4.4.1 software. Variables with more than 20% missing percentage were first excluded, and then Random Forest Multiple Interpolation was performed using the chained equation method to complete 5 iterations of data interpolation. Finally, the average of the results of the 5 iterations was calculated as the final filled data. Baseline data before and after interpolation were compared to assess the reliability of the filled data.

### 2.4 Statistical analysis

Data analysis in this study was done using SPSS 26.0 and R 4.4.1 software. Quantitative variables were described based on the results of the Shapiro–Wilk test: normally distributed variables were expressed as mean ± standard deviation (Mean ± SD), while non-normally distributed variables were presented as median (interquartile range) [M (P25, P75)]. For inter-group comparisons, normally distributed variables were analyzed using independent sample *t*-tests, non-normally distributed variables were analyzed using Mann–Whitney *U* tests, and categorical variables were analyzed using chi-square tests. The Least Absolute Shrinkage and Selection Operator (LASSO) regression was used to select all variables except metabolic and inflammatory indices, with the selected mortality-related variables included as covariates in the generalized linear model. Metabolic and inflammatory indices were grouped by quartiles, and trend tests were conducted using the group medians, adjusting for covariates selected by LASSO. Restricted cubic spline (RCS) analysis combined with multivariable logistic regression was used to explore the nonlinear relationship between metabolic and inflammatory indices and mortality risk. The optimal number of knots for the model was determined by comparing 3–7 knot models using the Akaike Information Criterion (AIC), adjusting for confounding factors, and results were presented as Odds Ratio(OR) and 95% confidence intervals (95% CI). The association of metabolic indices was assessed using partial correlation regression (Mantel test) to evaluate the matrix correlation between TyG and AIP.

The variable transformation rules were as follows: age was classified as <60, 60–74, and ≥75 years according to the World Health Organization criteria; SII was divided into five groups according to the sample percentile: < 399.23 × 10⁹/L (<P20 group), 399.23 × 10^9^/L−601.20 × 10^9^/L (P20-P40 group), 601.20 × 10^9^/L-892.50 × 10^9^/L (P40–P60 group), 892.50 × 10^9^/L-1539.58 × 10^9^/L (P60–P80 group), ≥1539.58 × 10^9^/L (≥P80 group). TyG and AIP were divided into low/high-value groups based on the median. The combined grouping of glucose and lipid metabolism included low glucose and low lipid group (TyG < P50 and AIP < P50), low glucose and high lipid group (TyG < P50 and AIP ≥ P50), high glucose and low lipid group (TyG ≥ P50 and AIP < P50), and high glucose and high lipid group (TyG ≥ P50 and AIP ≥ P50). Clinical indicators with *P* < 0.05 were screened by univariate analysis, and associations between groups of categorical variables were analyzed by chi-square test (association of SII quintile groups with mortality, and differences in the distribution of inflammatory indicators between metabolic subgroups). Multifactorial logistic regression models were analyzed using forced inclusion to include the screened variables in the analysis. Causal mediation analyses were performed using the R language “mediation” package, with TyG/AIP as the independent variable, death as the dependent variable, and SII as the mediator, adjusted for sex and age. All statistical analyses were performed using two-sided tests, with *P* < 0.05 as the threshold of significance.

## 3 Results

### 3.1 Patients with acute coronary syndrome

#### 3.1.1 Patient characteristics

A total of 3,861 patients diagnosed with ACS were included in this study, with an average age of 71.57 years, of which 2,795 were male (72.39%). After medical management, 3,655 patients survived, while 206 patients died. Analysis of mortality rates in different subgroups showed that STEMI patients had the highest mortality rate at 8.00%, while UA patients had the lowest at 0.90%. Age-based grouping revealed that patients aged ≥75 years had the highest mortality rate at 9.75%. In gender-based grouping, female patients had a higher mortality rate at 8.30%. Additionally, patients with a history of hypertension had a mortality rate of 5.70%, and those with diabetes had a mortality rate of 7.00%. Patients with a smoking history had a mortality rate of 3.50%, while those with an alcohol consumption history had a mortality rate of 3.10% (Table 1).

**Table 1:** Descriptive characteristics of patients with ACS stratified by mortality status (*N* = 3,861).

Variables	Death	Alive	*p* value
	*N* = 206	*N* = 3,655	
Type			*<0*.001***
NSTEMI	80 (7.00%)	1,060 (93.00%)	
STEMI	102 (8.10%)	1,156 (91.90%)	
UA	24 (1.64%)	1,439 (98.36%)	
Age	82.00 ± 10.04	70.98 ± 12.87	*<0*.001***
<60	8 (1.08%)	733 (98.92%)	*<0*.001***
60–74	26 (1.92%)	1,329 (98.08%)	
≥75	172 (9.75%)	1,593 (90.25%)	
Gender			*<0*.001***
Male	117 (4.20%)	2,678 (95.80%)	
Female	89 (8.30%)	997 (91.70%)	
Hypertension			*0*.*119*
No	52 (4.50%)	1,116 (95.50%)	
Yes	154 (5.70%)	2,539 (94.30%)	
Diabetes			*0*.002**
No	116 (4.50%)	2,453 (95.50%)	
Yes	90 (7.00%)	1,202 (93.00%)	
Smoking			*<0*.001***
No	150 (6.60%)	2,107 (93.40%)	
Yes	56 (3.50%)	1,548 (96.50%)	
Alcohol			*0*.009**
No	188 (5.70%)	3,098 (94.30%)	
Yes	18(3.10%)	557(96.90%)	

NSTEMI, non-ST-segment elevation myocardial infarction; STEMI, ST-segment elevation myocardial infarction; UA, unstable angina.

** *p* < 0.01.

*** *p* < 0.001.

In clinical indicator testing among ACS patients, significant differences were observed between deceased and surviving patients at admission regarding Low-Density Lipoprotein Cholesterol (LDL-C), TC, TG, creatinine (Crea), blood glucose, HDL-C, SII, and TyG, with statistical significance (*P* < 0.05). The LDL-C level in the deceased group (2.36 ± 0.87) was lower than that in the surviving group (2.61 ± 0.89). The TC level in the deceased group (4.03 ± 1.25) was also lower than that in the surviving group (4.29 ± 1.18). Furthermore, the median creatinine concentration in the deceased group [126 (89.75, 202.25)] was significantly higher than the median in the surviving group [82 (68, 103)], and the mean Crea comparison showed that the deceased group (10.44 ± 6.37) was higher than the surviving group (8.01 ± 3.92). The HDL-C level in the deceased group (1.01 ± 0.28) was lower than that in the surviving group (1.09 ± 0.27), while the median SII in the deceased group [1,135.86 (593.23, 2,784.61)] was significantly higher than that in the surviving group [716.62 (441.19, 1,287.06)]. The TyG level in the deceased group (9.20 ± 0.82) was higher than that in the surviving group (9.04 ± 0.72). Although the AIP in the deceased group (0.51 ± 0.26) was higher than that in the surviving group (0.49 ± 0.25), this difference did not reach statistical significance (Supplementary Table S1).

#### 3.1.2 LASSO regression for mortality risk factor selection and generalized linear model validation

**Figure 2:**
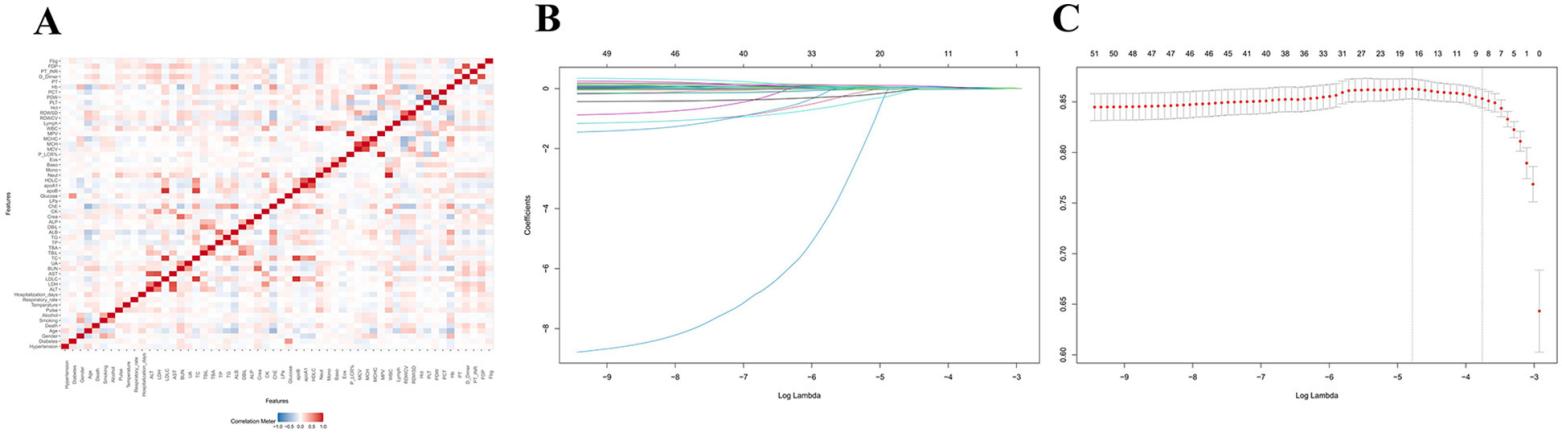
Feature screening and generalized linear modeling of death outcomes based on LASSO regression. **(A)** LASSO variable screening: penalized regression of candidate variables to automatically select variables highly correlated with mortality outcomes. **(B)** Key variable identification: nine statistically and clinically significant variables were finally identified. **(C)** Multivariate generalized linear regression: the screened variables were used as covariates to construct the GLM, which further quantified the effects of each variable on death outcomes and ensured the robustness and explanatory power of the model.

**Figure 3:**
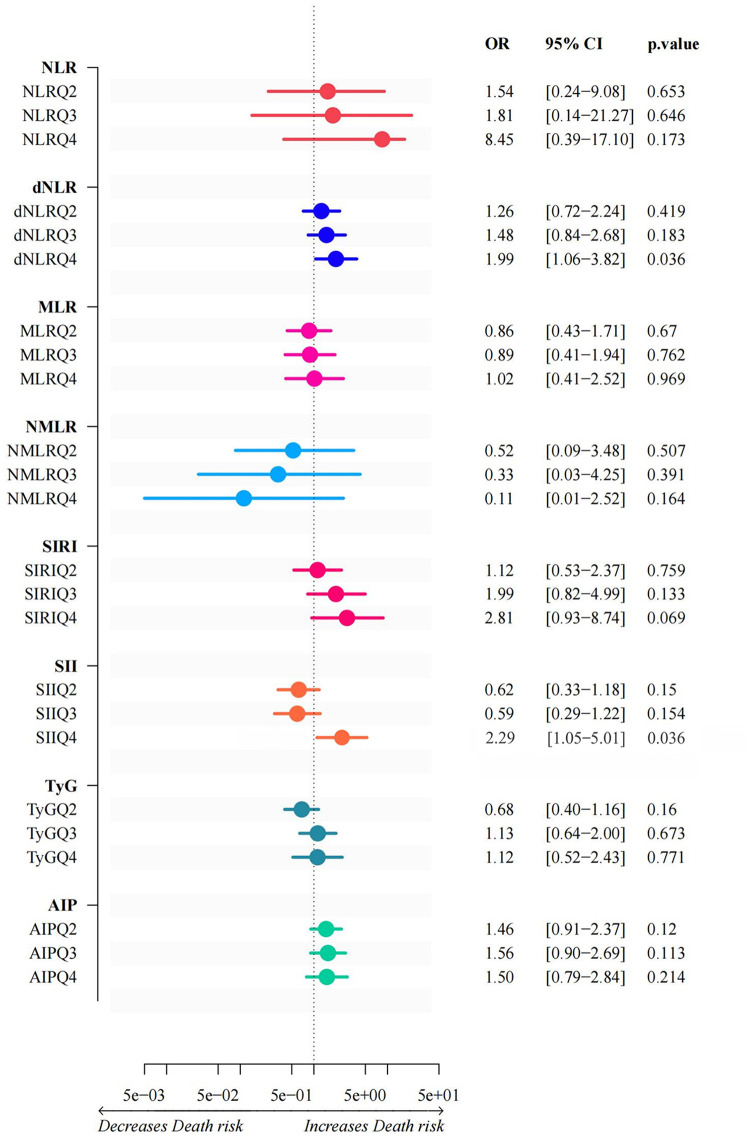
Generalized linear models of inflammation indices and glycolipid metabolism. This graph illustrates the results of a generalized linear model between inflammatory indices and glycolipid metabolism. The horizontal coordinates of each index represent the corresponding ratio, along with its 95% confidence interval and *p*-value. Depending on the shift direction of the OR value, the horizontal coordinate is between “Decreases Death risk” and “Increases Death risk”.

Figure 2 shows that after characterization of all variables (excluding metabolic and validation indicators) by the LASSO regression model, 9 independent predictor variables [Age, Sex, Diabetes Mellitus, Smoking Status, Heart Rate, Albumin (ALB), Blood Urea Nitrogen (BUN), Cholinesterase (ChE), D-Dimer Fragment (D-Dimer)] were identified as significantly associated with CVD mortality outcomes (Supplementary Table S2), with non-zero regression coefficients for each variable. In subsequent models (generalized linear models, restricted cubic spline analysis, mediation analysis) as well as in ACS and AMI subgroup analyses, the same set of covariates was consistently used. Based on the screening results, generalized linear regression models were further constructed to assess their independent effects on mortality outcomes. As shown in Figure 3, elevated dNLR was significantly and positively associated with the risk of death (highest vs. lowest quartile: OR = 1.99, 95% CI 1.06–3.82, *P* = 0.036), the highest quartile of SII levels was negatively associated with the risk of death (OR = 2.29, 95% CI 1.05–5.01, *P* = 0.036). Other variables did not reach statistical significance (*P* ≥ 0.05), although they tended to be positively correlated with mortality.

#### 3.1.3 Nonlinear relationship analysis between inflammatory and metabolic indices and mortality

**Figure 4:**
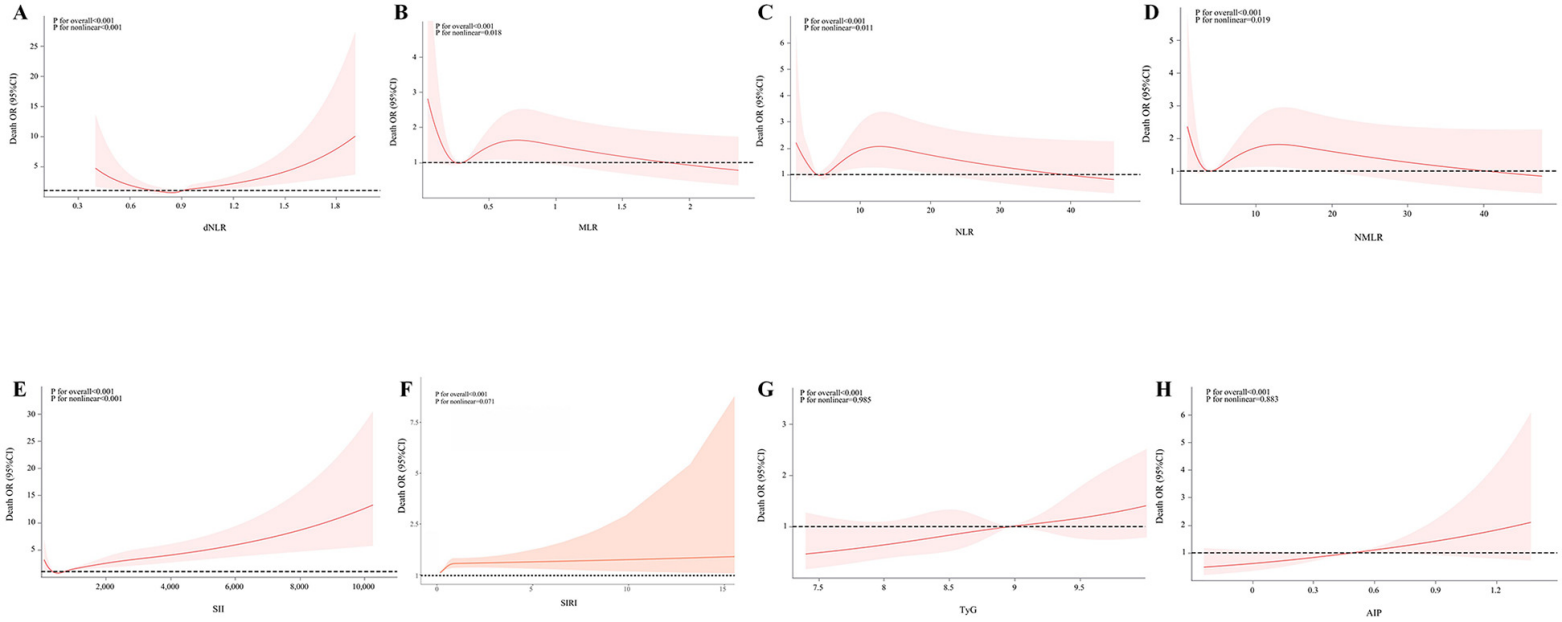
Associations of inflammatory indicators and glycolipid metabolism indicators with mortality in ACS patients. The associations between MLR, NLR, NMLR, SII, SIRI, TyG, and AIP and the mortality in the population of acute coronary syndrome patients were analyzed by evaluating the overall significance and nonlinear significance. The *P* values for the overall effect and nonlinearity were calculated. The solid lines in the corresponding charts represent the estimated relationships, and the shaded areas indicate the 95% confidence intervals.

Figure 4 reveals the complex nonlinear associations between inflammatory indices and metabolic indices with mortality in acute coronary syndrome patients. Statistical analysis showed that systemic inflammatory markers dNLR and SII exhibited significant nonlinear associations with mortality (*P* overall < 0.001, *P* non-linear < 0.001), with their dose-response curves displaying a “U-shaped” pattern, indicating that both low and high concentrations may increase mortality risk. Similarly, MLR, NLR, and NMLR also demonstrated significant nonlinear associations (*P* overall < 0.001, *P* non-linear < 0.005), but exhibited a unique inverted U-shaped dose-response characteristic, where mortality risk was higher at low concentrations and gradually decreased with increasing concentrations. Notably, among the metabolic indices, TyG and AIP showed significant linear associations with mortality (*P* overall < 0.001), but no significant nonlinear effects were detected (*P* non-linear > 0.005), with mortality risk monotonically increasing with concentration. The analysis of the SIRI index indicated that despite the overall association being statistically significant (*P* overall < 0.001), its nonlinear effect did not reach significance (*P* non-linear = 0.071), although the curve's “gradual decline” trend suggests that higher SIRI values may be associated with lower mortality risk.

#### 3.1.4 Correlation analysis of inflammatory indices and metabolic indices

**Figure 5:**
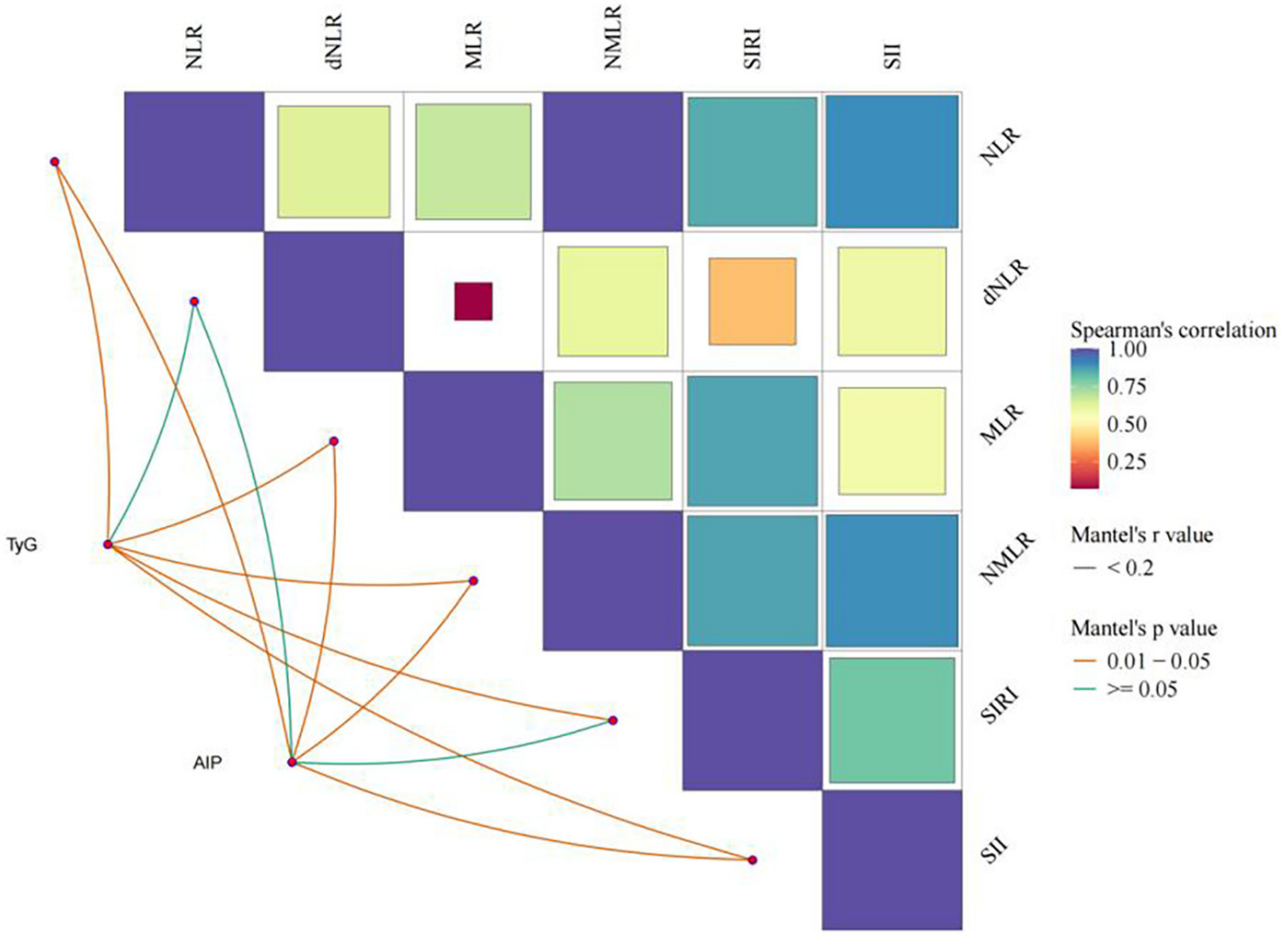
Analysis of the association between inflammatory indicators and glycolipid metabolism.

Figure 5 indicates a certain degree of correlation between inflammatory indices, with Spearman correlation coefficients represented by the size and color intensity of squares: larger squares and darker colors indicate stronger correlations. The correlation of metabolic indices was assessed through partial correlation regression analysis, with yellow lines indicating statistically significant associations (*P* < 0.05). TyG was significantly correlated with all inflammatory indices except dNLR, while AIP was significantly associated with all inflammatory indices except dNLR and SIRI.

#### 3.1.5 Association analysis of SII grouping with glycolipid metabolism distribution and mortality

Based on the literature review and considering the comprehensive advantages of SII in reflecting systemic inflammatory response and immune status, as well as its predictive value for cardiovascular patients' prognosis in multiple studies (28), SII was ultimately selected as the representative indicator of patients' inflammatory status for in-depth analysis.

Statistical analysis indicated that the distribution differences of metabolic groups among SII groups were statistically significant (*P* < 0.001). In ACS patients, the SII < P20 group had the lowest proportion of high glucose and low lipid patients (6.21%, 48/773), while the high glucose and high lipid patients had the highest proportion (40.88%, 316/773). The SII P20–P40 group was primarily composed of low glucose and low lipid patients (41.32%, 319/772), with the lowest proportion of high glucose and low lipid patients (6.99%, 54/772). In the SII P40–P60 group, the proportion of high glucose and low lipid patients was the lowest (11.01%, 85/772), while the proportion of high glucose and high lipid patients was the highest (38.86%, 300/772). In the SII P60–P80 group, the proportion of low glucose and high lipid patients further decreased to 8.29% (64/772), with high glucose and high lipid patients still dominating (40.16%, 310/772) (Table 2).

**Table 2:** Inflammation levels in patients with ACS with different levels of glucose and lipid metabolism (*N* = 3,861).

SII	Low-glucose-low-lipid level group	Low-glucose-high-lipid level group	High-glucose-low-lipid level group	High-glucose-high-lipid level group	*P* Value
<P20	302 (39.07%)	107 (13.84%)	48 (6.21%)	316 (40.88%)	*<0.001* ***
P20–P40	319 (41.32%)	87 (11.27%)	54 (6.99%)	312 (40.41%)	
P40–P60	296 (38.34%)	91 (11.79%)	85 (11.01%)	300 (38.86%)	
P60–P80	284 (36.79%)	64 (8.29%)	114 (14.77%)	310 (40.16%)	
≥P80	306 (39.64%)	74 (9.59%)	122 (15.80%)	270(34.97%)	

SII, systemic immune-inflammation index.

*** *p* < 0.001.

Notably, the SII ≥ P80 group exhibited a different trend, with the highest proportion of low glucose and low lipid patients (39.64%, 306/772), while the proportion of low glucose and high lipid patients was the lowest (9.59%, 74/772) (Table 3).

**Table 3:** The chi-square test for the relationship between SII and mortality in patients with ACS (*N* = 3,861).

SII	Death	Alive	*p* value
	*N* = 206	*N* = 3,655	
*<*P20	31 (4.01%)	742 (95.99%)	*<0.001* ***
P20–P40	20 (2.59%)	752 (97.41%)	
P40–P60	26 (3.37%)	746 (96.63%)	
P60–P80	43 (5.57%)	729 (94.43%)	
≥P80	86 (11.14%)	686 (88.86%)	

SII, systemic immune-inflammation index.

*** *p* < 0.001.

#### 3.1.6 Multivariable logistic regression analysis of factors influencing patient mortality

**Figure 6:**
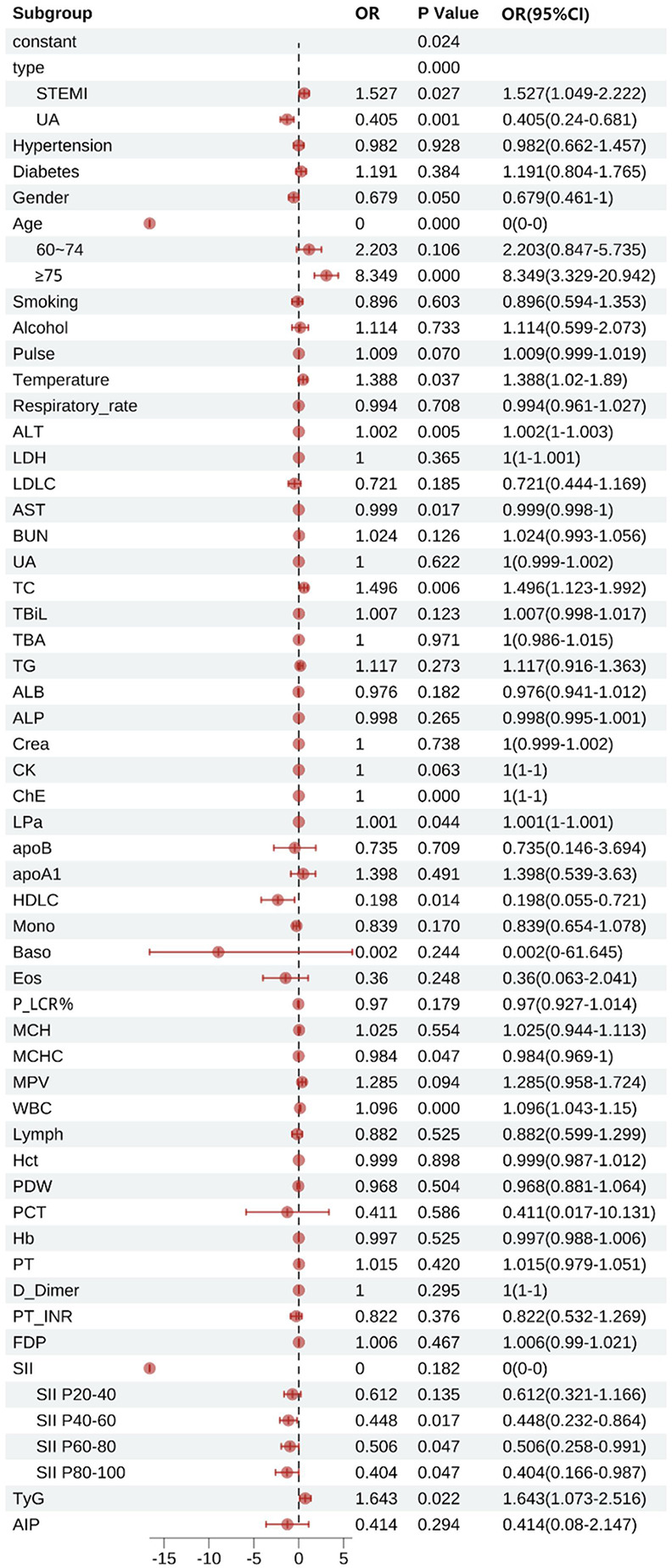
Multifactorial logistic regression of factors influencing death in patients with acute coronary syndromes. STEMI, ST-segment elevation myocardial infarction, UA, unstable angina; Pulse, pulse rate; ALT, alanine aminotransferase; LDH, lactate dehydrogenase; LDLC, low-density lipoprotein cholesterol; AST, aspartate aminotransferase; BUN, blood urea nitrogen; UA, uric acid; TC, total cholesterol; TBiL, total bilirubin; TBA, total bile acid; TG, triglyceride; ALB, albumin; ALP, alkaline phosphatase; Crea, creatinine; CK, creatine kinase; ChE, cholinesterase; LPa, lipoprotein(a); apoB, apolipoprotein B; apoA1, apolipoprotein A1; HDLC, high-density lipoprotein cholesterol; Mono, monocytes; Baso, basophils; Eos, eosinophils; P_LCR%, platelet large cell ratio percentage; MCH, mean corpuscular hemoglobin; MCHC, mean corpuscular hemoglobin concentration; MPV, mean platelet volume; WBC, white blood cells; Lymph, lymphocytes; Hct, hematocrit; PDW, platelet distribution width; PCT, plateletcrit; Hb, hemoglobin; D_Dimer, D-dimer; PT_INR, prothrombin time international normalized ratio; FDP, fibrin degradation products; SII, systemic immune-inflammation index; TyG, triglyceride-glucose index; AIP, atherogenic index of plasma.

Figure 6 shows that type, age, temperature, Alanine Aminotransferase(ALT), AST, TC, ChE, lipoprotein a(LPa), HDL-C, mean corpuscular hemoglobin concentration(MCHC), WBC, SII, and TyG were statistically significant factors associated with patient mortality (*P* < 0.05). In terms of disease classification, the mortality risk for STEMI patients was 1.527 times that of NSTEMI patients (95% CI 1.049–2.222), while UA patients had a mortality risk of only 0.405 times that of NSTEMI patients (95% CI 0.240–0.681). The mortality risk for patients with hypertension was not significantly different from that of patients without hypertension (OR = 0.982, 95% CI 0.662–1.457), while the mortality risk for patients with diabetes was slightly higher than that for patients without diabetes, but did not reach statistical significance (OR = 1.191, 95% CI 0.804–1.765). Gender analysis showed that male patients had a 32.1% lower mortality risk than female patients (OR = 0.679, 95% CI 0.461–1.000). In age stratification analysis, the mortality risk for patients aged 60–74 years was 2.203 times that of patients aged <60 years (95% CI 0.847–5.735), while the mortality risk for patients aged ≥75 years significantly increased to 8.349 times that of patients aged <60 years (95% CI 3.329–20.942). The influence of smoking and alcohol consumption history on mortality risk did not reach statistical significance (smoking history OR = 0.896, 95% CI 0.594–1.353; alcohol consumption history OR = 1.114, 95% CI 0.599–2.073).

Subgroup analyses of SII showed that the mortality risk for the P20–P40 group was 0.612 times that of patients in the P20 group (95% CI 0.321–1.166), the mortality risk for the P40–P60 group was 0.448 times that of patients in the P20 group (95% CI 0.232–0.864), and the mortality risk for the P60–P80 group was 0.506 times that of patients in the P20 group (95% CI 0.258–0.991), while the mortality risk for patients in the P80 group dropped to 0.404 times that of patients in the P20 group (95% CI 0.166–0.987).

In laboratory indicators, for every unit increase in TC, the mortality risk increased by 49.6% (OR = 1.496, 95% CI 1.123–1.992). HDL-C demonstrated a significant protective effect (OR = 0.198, 95% CI 0.055–0.721). For every increase of 1 × 10⁹/L in WBC, the mortality risk increased by 9.6% (OR = 1.096, 95% CI 1.043–1.150). Additionally, TyG was also an independent predictor of mortality risk: for every unit increase in TyG, the mortality risk increased by 64.3% (OR = 1.643, 95% CI 1.073–2.516). However, LDL-C (OR = 0.721, 95% CI 0.444–1.169), TG (OR = 1.117, 95% CI 0.916–1.015), apolipoprotein B (apoB) (OR = 0.735, 95% CI 0.146–3.694), apolipoprotein A1 (apoA1) (OR = 1.398, 95% CI 0.539–3.630), hematocrit (Hct) (OR = 0.999, 95% CI 0.987–1.012), and AIP (OR = 0.414, 95% CI 0.080–2.147) did not show significant associations.

### 3.2 Subgroup of patients with acute myocardial infarction

#### 3.2.1 Patient characteristics

A total of 2,398 patients with confirmed infarction were included in the subgroup analysis, with a mean age of 71.00 years. After treatment, 2,216 patients survived and 182 died. The analysis showed that the mortality rate was 8.1% in STEMI patients and 7.0% in NSTEMI patients, with no statistically significant difference (*P* = 0.317). Among the age subgroups, patients aged ≥75 years old had the highest mortality rate of 13.5%. In contrast, patients <60 years old had the lowest mortality rate of 1.3%, and both differences were statistically significant (*P* < 0.001). Gender analysis showed that female patients had a mortality rate of 11.5%, which was significantly higher than that of male patients at 6.1% (*P* < 0.001). The mortality rate of patients with combined hypertension was 8.3%, which was slightly higher than 6.2% of patients without combined hypertension, but the difference was not statistically significant (*P* = 0.083). The mortality rate of patients with combined diabetes was 10.5%, which was significantly higher than that of patients without combined diabetes, which was 6.1% (*P* < 0.001). Patients with a smoking history had a mortality rate of 4.7%, significantly lower than that of patients without a smoking history at 9.7% (*P* < 0.001). Meanwhile, patients with a history of alcohol consumption had a mortality rate of 3.8%, which was significantly lower than that of 8.3% in patients without a history of alcohol consumption (*P* = 0.002). The results indicate that age, gender, diabetes, smoking history, and drinking history are important factors affecting mortality in AMI patients. Among them, patients with advanced age, female gender, and diabetes are associated with higher mortality risk, while smoking and drinking history may be related to lower mortality rates (Table 4).

**Table 4:** Descriptive characteristics of patients with AMI stratified by mortality status (*N* = 2,398).

Variables	Death	Alive	*p* value
	*N* = 182	*N* = 2,216	
Type			*<0*.001***
NSTEMI	80 (7%)	1,060 (93%)	
STEMI	102 (8.1%)	1,156 (91.9%)	
Age	82 ± 10.04	70.98 ± 12.86	*<0*.001***
<60	7 (1.3%)	517 (98.7%)	*<0*.001***
60–74	23 (3.1%)	724 (96.9%)	
≥75	152 (13.5%)	975 (86.5%)	
Gender	105 (6.1%)	1,626 (93.9%)	*<0*.001***
Male	77 (11.5%)	590 (88.5%)	
Female			
Hypertension	48 (6.2%)	728 (93.8%)	*0*.*119*
No	134 (8.3%)	1,488 (91.7%)	
Yes			
Diabetes	99 (6.1%)	1,511 (93.9%)	*0*.002**
No	83 (10.5%)	705 (89.5%)	
Yes			
Smoking	134 (9.7%)	1,250 (90.3%)	*<0*.001***
No	48 (4.7%)	966 (95.3%)	
Yes			
Alcohol	168 (8.3%)	1,864 (91.7%)	*0*.009**
No	14 (3.8%)	352 (96.2%)	
Yes	80(7%)	1,060(93%)	

NSTEMI, non-ST-segment elevation myocardial infarction; STEMI, ST-segment elevation myocardial infarction.

** *p* < 0.01.

*** *p* < 0.001.

In the clinical indexes tested in patients with acute myocardial infarction, there were significant differences in several indexes between the deceased patients and the surviving patients. The LDL-C level in the deceased patients (2.38 ± 0.85) was significantly lower than that in the surviving patients (2.74 ± 0.89, *P* < 0.001), and the TC level in the deceased patients (4.04 ± 1.21) was also significantly lower than that in the surviving patients (4.41 ± 1.17, *P* < 0.001). The difference in TG levels between deceased patients (1.63 ± 1.49) and surviving patients (1.75 ± 1.45) was not statistically significant (*P* = 0.273). For creatinine levels, the median [127 (93, 205)] was significantly higher in deceased patients than in surviving patients [84 (68, 107)] (*P* < 0.001). Additionally, the HDL-C level in deceased patients (1.00 ± 0.27) was significantly lower than that in surviving patients (1.08 ± 0.26, *P* < 0.001), while the WBC level in deceased patients (11.78 ± 6.38) was significantly higher than that in surviving patients (9.27 ± 4.09, *P* < 0.001). The lymphocyte level in deceased patients (1.24 ± 0.70) was significantly lower than that in surviving patients (1.45 ± 0.69, *P* < 0.001), but the RDW-CV level in deceased patients (14.17 ± 2.02) was significantly higher compared to surviving patients (13.30 ± 1.15, *P* < 0.001). The SII in deceased patients [1,190.92 (658.64, 2,842.86)] was significantly higher than that in surviving patients [869.51 (533.45, 1,566.01)] (*P* < 0.001), and the TyG index in deceased patients (9.22 ± 0.81) was also significantly higher than that in surviving patients (9.09 ± 0.73, *P* = 0.017). As for AIP levels, although they were higher in dead patients (0.51 ± 0.26) than in surviving patients (0.50 ± 0.26), the difference was not statistically significant (*P* = 0.689) (Supplementary Table S3).

#### 3.2.2 Association analysis of SII grouping with glycolipid metabolism distribution and mortality

In AMI patients, the differences in the distribution of metabolic groups among SII groups were statistically significant (*P* < 0.001). In the SII < P20 group, the proportion of high glucose and low lipid patients was the lowest (7.10%, 23/324), while the proportion of high glucose and high lipid patients was the highest (44.75%, 145/324). The SII P20–P40 group was primarily composed of low glucose and low lipid patients (41.41%, 164/396), with the lowest proportion of high glucose and low lipid patients (5.56%, 22/396). In the SII P40–P60 group, the proportion of low glucose and high lipid patients was the lowest (10.90%, 52/477), while the proportion of high glucose and high lipid patients was the highest (40.04%, 191/477). In the SII P60–P80 group, the proportion of low glucose and high lipid patients further decreased to 8.21% (46/560), with high glucose and high lipid patients still dominating (43.04%, 241/560). Notably, in the SII ≥ P80 group, there was a different trend, with the highest proportion of low glucose and low lipid patients (37.91%, 243/641), while the proportion of low glucose and high lipid patients was the lowest (9.36%, 60/641) (Table 5).

**Table 5:** Inflammation levels in patients with AMI with different levels of glucose and lipid metabolism (*N* = 2,398).

SII	Low-glucose-low-lipid level group	Low-glucose-high-lipid level group	High-glucose-low-lipid level group	High-glucose-high-lipid level group	*P* Value
<P20	108 (33.33%)	48 (14.81%)	23 (7.10%)	145 (44.75%)	*<0.001* ***
P20–P40	164 (41.41%)	47 (11.87%)	22 (5.56%)	163 (41.16%)	
P40–P60	178 (37.32%)	52 (10.90%)	56 (11.74%)	191 (40.04%)	
P60–P80	188 (33.57%)	46 (8.21%)	85 (15.18%)	241 (43.04%)	
≥P80	243 (37.91%)	60 (9.36%)	109 (17.00%)	229 (35.73%)	

SII, systemic immune-inflammation index.

*** *p* < 0.001.

Among patients with AMI, there were significant differences in mortality rates among different SII subgroups (*P* < 0.001). The SII P20–P40 group of patients had the lowest number of deaths at 15 (3.79%), while the SII ≥ P80 group had the highest number of deaths at 80 (12.48%) (Table 6).

**Table 6:** The chi-square test for the relationship between SII and mortality in patients with AMI (*N* = 2,398).

SII	Death	Alive	*p* value
	*N* = 182	*N* = 2,216	
*<*P20	25 (7.72%)	299 (92.28%)	*<0.001* ***
P20–P40	15 (3.79%)	381 (96.21%)	
P40–P60	23 (4.82%)	454 (95.18%)	
P60–P80	39 (6.96%)	521 (93.04%)	
≥P80	80 (12.48%)	561 (87.52%)	

SII, systemic immune-inflammation index.

*** *p* < 0.001.

#### 3.2.3 Multivariable logistic regression analysis of factors influencing patient mortality

**Figure 7:**
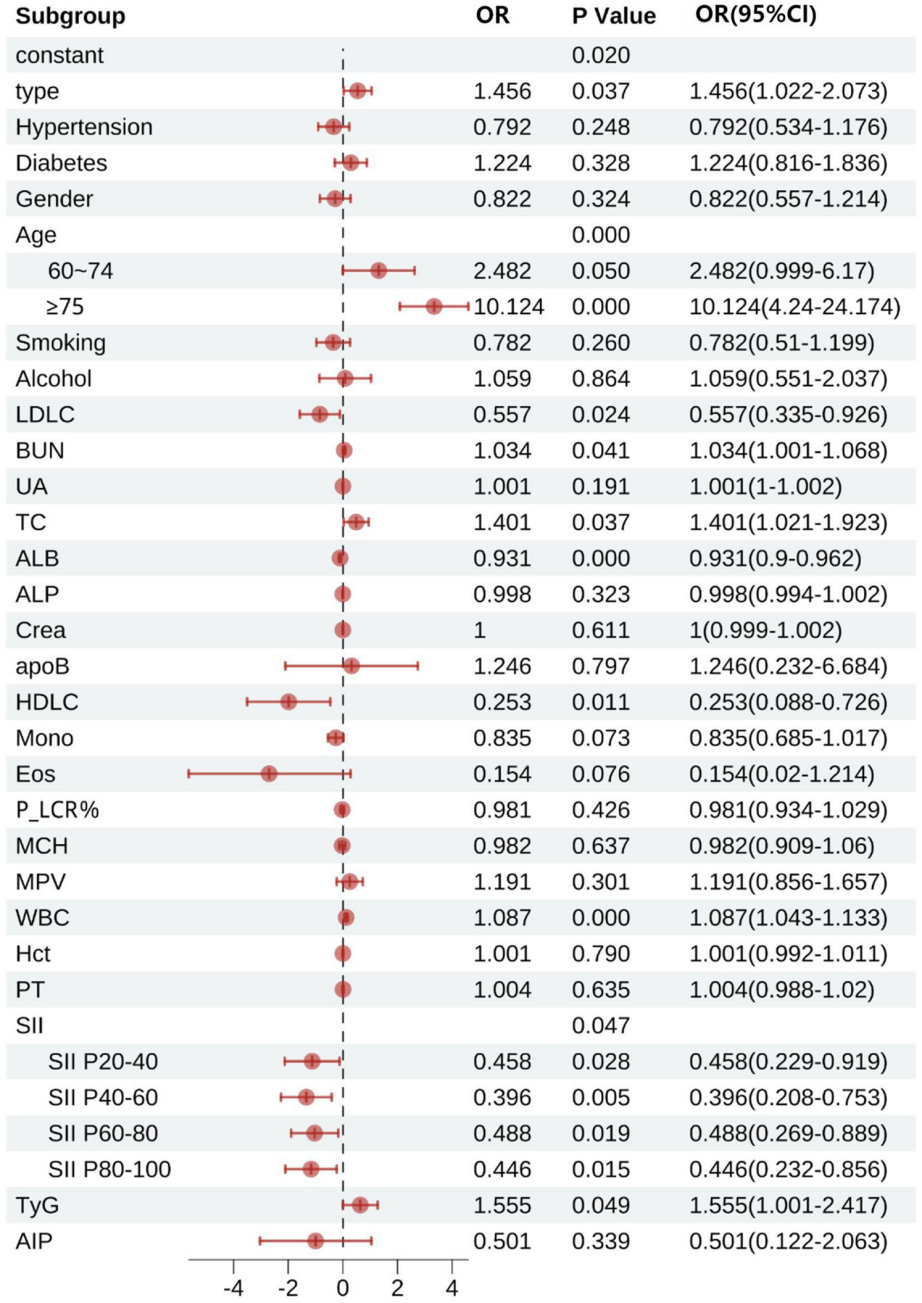
Multifactorial logistic regression of factors influencing death in patients with acute myocardial infarction. LDLC, low-density lipoprotein cholesterol; BUN, blood urea nitrogen; UA, uric acid; TC, total cholesterol; ALB, albumin; ALP, alkaline phosphatase; Crea, creatinine; apoB, apolipoprotein B; HDLC, high-density lipoprotein cholesterol; Mono, monocytes; Eos, eosinophils; P_LCR%, platelet large cell ratio percentage; MCH, mean corpuscular hemoglobin; MPV, mean platelet volume; WBC, white blood cells; Hct, hematocrit; PT, prothrombin time; SII, systemic immune-inflammation index; SII P20–40, SII for 20 to 40 percent; SII P40–60, SII for 40 to 60 percent; SII P60–80, SII for 60 to 80 percent; SII P80–100, SII for 80 to 100 percent; TyG, triglyceride-glucose index; AIP, atherogenic index of plasma.

Figure 7 demonstrates that type, age, LDL-C, BUN, TC, ALB, HDL-C, TyG, WBC, and SII were all statistically significant factors associated with patient mortality (all *P* < 0.05). In terms of disease classification, the mortality risk for STEMI patients was 1.456 times that of NSTEMI patients (95% CI: 1.022–2.073). The mortality risk for patients with hypertension was not significantly different from that of patients without hypertension (OR = 0.792, 95% CI: 0.534–1.176). In contrast, patients with diabetes had a slightly higher mortality risk compared to those without diabetes (OR = 1.224, 95% CI: 0.816–1.836), but this did not reach statistical significance. Gender analysis indicated that male patients had a 17.8% lower mortality risk than female patients (OR = 0.822, 95% CI: 0.557–1.214).In age stratification, patients aged 60–74 years had a mortality risk that was 2.482 times that of patients aged <60 years (95% CI: 0.999–6.170), while those aged ≥75 years had a significantly higher mortality risk of 10.124 times that of patients aged <60 years (95% CI: 4.240–24.174). The effects of smoking and drinking histories on mortality risk did not reach statistical significance (smoking history OR = 0.782, 95% CI: 0.510–1.200; alcohol consumption history OR = 0.789, 95% CI: 0.551–2.037).

Subgroup analyses of SII revealed that compared to the < P20 group, the mortality risk for the P20–40 group was reduced by 54.2% (OR = 0.458, 95% CI: 0.229–0.919), the risk for the P40–60 group was reduced by 60.4% (OR = 0.396, 95% CI: 0.208–0.753), the risk for the P60–80 group was reduced by 51.2% (OR = 0.488, 95% CI: 0.269–0.889), and the risk for the ≥P80 group was further reduced by 55.4% (OR = 0.446, 95% CI: 0.232–0.856).

In terms of laboratory indicators, for every 1 mmol/L increase in TC, the mortality risk increased by 40.1% (OR = 1.401, 95% CI: 1.021–1.923), while HDL-C showed a significant protective effect (OR = 0.280, 95% CI: 0.138–0.576). For every increase of 1 × 10⁹/L in WBC, the mortality risk increased by 8.7% (OR = 1.087, 95% CI: 1.043–1.133). Additionally, TyG was identified as an independent risk factor, with each unit increase in TyG associated with a 55.5% increase in mortality risk (OR = 1.555, 95% CI: 1.001–2.417). Other indicators, such as LDL-C (OR = 0.557, 95% CI: 0.335–0.926) and ALB (OR = 0.931, 95% CI: 0.9–0.962), were associated with a risk reduction, while BUN (OR = 1.034, 95% CI: 1.001–1.068) showed a slight increase in risk. AIP (OR = 0.501, 95% CI: 0.122–2.063) did not demonstrate a significant association.

### 3.3 Mediating role of inflammation in the relationship between metabolic disorders and mortality

**Figure 8:**
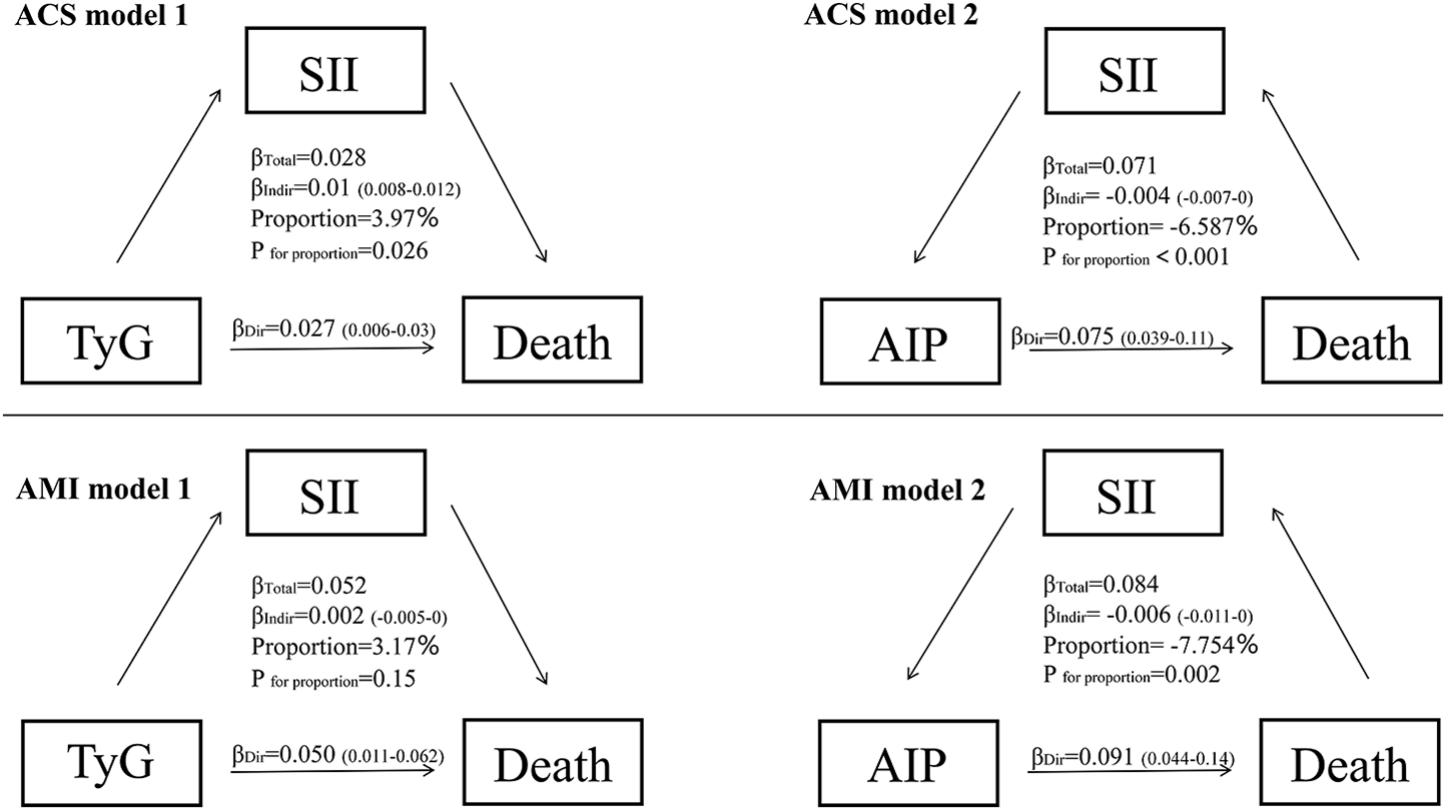
Mediation analysis for the associations between TyG, AIP, SII and death in ACS and AMI patients. ACS, acute coronary syndrome, AMI, acute myocardial infarction; SII, systemic immune-inflammation index; TyG, triglyceride-glucose index; AIP, atherogenic index of plasma.

Figure 8 demonstrates that the impact of TyG and AIP on mortality in ACS and AMI patients is primarily mediated through direct effects. In contrast, SII exhibits distinct mechanisms of mediation in its relationship with TyG and AIP, particularly demonstrating a suppressive effect in the context of AIP.

## 4 Discussion

In patients with ACS and AMI, the impact of glucose metabolism disorders, indicated by the TyG index, on mortality risk is primarily due to direct effects rather than inflammation-mediated pathways. Multivariable regression analysis shows a significant positive correlation between the TyG index and mortality risk (ACS group: OR = 1.643, 95% CI 1.073–2.516; AMI subgroup: OR = 1.555, 95% CI: 1.001–2.417, both *P* < 0.05) (Figures 3, 8). A significant linear dose-response relationship between TyG index and mortality was further confirmed by RCS analysis (ACS group: *p* overall < 0.001, *p* nonlinear = 0.985; AMI subgroup: *p* overall < 0.001, *p* nonlinear = 0.767) (Figure 4G, Supplementary Figure S1B). Mediation analysis further confirmed the weak mediating contribution of SII in this association: the average mediating effect accounted for only 3.97% in the ACS group (*p* = 0.026) and was not statistically significant in the AMI group (*p* = 0.15). In contrast, the direct effect was statistically significant (both *p* < 0.001) (Figure 8). This finding corroborates with the direct pathogenic mechanism of acute hyperglycemia revealed by basic studies: elimination of ischemic preadaptive protection by closing ATP-dependent potassium channels in cardiomyocytes (29) and promotion of platelet-dependent thrombosis and microcirculatory disturbances (30, 31).

From a pathophysiological perspective, the “stress hyperglycemia survival hypothesis” proposed by Marik and Bellomo provides a theoretical framework for this phenomenon. The activation of the sympathoadrenal axis during the acute phase prioritizes energy supply to immune and neuronal cells through hepatic gluconeogenesis and insulin resistance (32). This process is accompanied by an upregulation of Glucose Transporter Type 1 expression to enhance glucose transport (33), resulting in a bidirectional regulation of immune function by blood glucose levels. The 7.8–10.0 mmol/L range enhances neutrophil bactericidal capacity, whereas a level exceeding 11.1 mmol/L leads to immunosuppression (34). Notably, the anti-inflammatory effect of glucocorticoids in the acute phase by inhibiting the Nuclear Factor kappa-light-chain-enhancer of activated B cells pathway (32) may weaken the chronic association of glucose metabolism disorders with inflammatory responses, which provides an explanation for the weak contribution of the SII-mediated pathway in the current study. This provides a mechanistic basis to explain the weak contribution of the SII-mediated pathway in this study.

In contrast to the single dominant pathway observed in glucose metabolism disorders, this study revealed a complex mechanistic action of lipid metabolism disorders, as characterized by the AIP index, on mortality risk in patients with ACS and AMI. Multifactorial logistic regression showed that AIP was not directly statistically associated (*P* > 0.05) with the risk of death in both ACS (OR = 0.414, 95% CI 0.080–2.147) and AMI (OR = 0.501, 95% CI 0.122–2.063) patients (Figures 3, 8). However, RCS analysis showed a significant linear dose-response relationship between AIP and mortality (ACS group: *P*-overall < 0.001, *P*-nonlinear = 0.883; AMI subgroup: *P*-overall < 0.001, *P*-nonlinear = 0.846) (Figure 4H, Supplementary Figure S1C), suggesting that elevated AIP may increase risk through cumulative effects not captured by the nonlinear model. Causal mediation analysis further resolved this paradox: the total effect of AIP on death was significantly positive (*β* = 0.071 in the ACS group and *β*=0.084 in the AMI subgroup, both *P* < 0.001), but its effect was decomposed by a bidirectional pathway—the Average Direct Effect (ADE) was dominant (ADE = 0.07567 in the ACS group and ADE = 0.09103 in the AMI subgroup, both *P* < 0.001). This suggests that each 1-unit elevation in AIP directly increased the risk of death by 7.57% in ACS patients and 9.10% in AMI patients. In contrast, the indirect effect via SII was negative (Average Causal Mediation Effect(ACME) = −0.00468 in the ACS group and ACME = −0.00655 in the AMI subgroup, both *P* ≤ 0.002), with mediation ratios of −6.59% in the ACS group and −7.75% in the AMI subgroup. This suggests that SII may partially counteract the direct harm of AIP by inhibiting inflammatory pathways (Figure 8). The “hazard-vicarious” mechanism is particularly prominent in AMI patients, manifesting as increased ADE intensity and a higher proportion of negative mediation. This supports the robustness of the conclusion that AIP elevates mortality risk via non-inflammatory pathways across diverse populations.

The direct harmful effect of AIP is closely related to its atherogenic lipid profile (high TG/low HDL-C ratio). Excess free fatty acids deposited within cardiomyocytes can activate lipotoxic signaling pathways, promoting cardiomyocyte apoptosis and ventricular remodeling (35). Meanwhile, Low HDL-C levels diminish cholesterol efflux capacity and anti-inflammatory/antioxidant activity, which is particularly pronounced during acute coronary events (36). Animal studies further indicate that in the early phase after myocardial infarction (within 24 h), the compensatory mechanism relies heavily on fatty acid oxidation for energy, accounting for over 70%. However, this reliance may lead to reduced ATP synthesis and lipotoxic injury due to ongoing lipid overload (37). This results in the establishment of a biphasic dynamic pattern of lipid metabolic imbalance.

Notably, the indirect protective effect of AIP mediated through SII suggests the complex interplay between lipid metabolism and inflammation. Hypertriglyceridemia may induce anti-inflammatory phenotypic polarization of monocytes and inhibit the release of pro-inflammatory factors through activation of the Peroxisome Proliferator-Activated Receptor Gamma(PPAR-γ) pathway (38). Furthermore, lipid mediators such as sphingosine-1-phosphate (S1P) and n-3 polyunsaturated fatty acids (n-3 PUFAs) can promote inflammation resolution (39). However, the AIP index [log(TG/HDL-C)] may underestimate their potential protective value because it fails to capture such functional dynamic changes.

In summary, elevated AIP primarily drives mortality risk through non-inflammatory pathways such as lipotoxic myocardial injury and metabolic imbalance. While the effect of inflammatory suppression (mediated through SII) is insufficient to reverse the overall risk. Similar to glucose metabolism disorders, lipid metabolism disorders are also centered on direct pathological effects. The key difference lies in the fact that lipid metabolism disorders reveal a non-linear association within the metabolism-inflammation network through the weak protective effects mediated by SII, whereas inflammatory pathways in glucose metabolism disorders exhibit almost no intermediary role. This finding highlights the critical role of lipid metabolism management in the secondary prevention of acute coronary events and suggests prioritizing interventions targeting non-inflammatory-dependent pathological mechanisms (40).

In the comprehensive assessment of inflammatory marker selection criteria, this study selected the Systemic Immune-Inflammation Index (SII) as the core inflammatory evaluation metric. This selection was based on its dual advantages in reflecting systemic inflammatory response and immune homeostasis (28), along with its independent predictive efficacy for cardiovascular outcomes. After identifying a nonlinear association between SII and prognosis in the overall ACS cohort, the study performed in-depth verification of a contradictory phenomenon observed in the preliminary analysis of the AMI subgroup. While univariate analysis showed increased mortality in the high SII group (≥P80), multivariate adjustments revealed a protective effect (Figure 7). With the RCS model, the study established a significant U-shaped nonlinear relationship between SII and mortality risk in AMI patients (nonlinear test *P* < 0.001) (Supplementary Figure S1A). Notably, this U-shaped association pattern is highly consistent with the nonlinear trend observed in the overall ACS cohort (nonlinear test *P* < 0.001). Based on model-predicted estimates, both low SII levels (<450 × 10^9^/L) and high SII levels (>900 × 10^9^/L) were associated with increased risk, while moderate levels (450–900 × 10^9^/L) corresponded to the lowest risk. Compared to traditional fixed grouping methods, RCS effectively reduced reference group selection bias and effect dilution through continuous modeling. As a result, this method captured the dynamic biological effects of SII more accurately.

Mechanistically, neutrophil and platelet deficiencies in low SII state impair inflammatory clearance, which leads to myocardial necrotic tissue retention (41). The relative dominance of lymphocytes may excessively suppress the necessary inflammatory repair during the acute phase. This phenomenon is particularly pronounced in frail or cachectic patients, whose state of immune paralysis exacerbates cardiovascular decompensation (42). At moderate SII levels, neutrophils clear damaged myocardial debris, platelets maintain inflammatory homeostasis, and lymphocytes inhibit excessive monocyte/macrophage infiltration (43, 44). These three cell types cooperatively regulate the repair-fibrosis balance. At high SII levels, abnormally activated neutrophils release pro-inflammatory factors and reactive oxygen species through Neutrophil Extracellular Trap Formation (45), while platelet-leukocyte aggregates exacerbate microcirculatory dysfunction (46). This ultimately leads to severe complications such as expanded myocardial edema and acute heart failure (47), increasing mortality rates.

This U-shaped association fundamentally reflects the bidirectional immune regulatory mechanism of the body in response to acute cardiovascular events: a moderate inflammatory response is essential for tissue repair, while both excessive activation and suppression can disrupt homeostasis. This suggests the need for clinical strategies to optimize anti-inflammatory treatment through sequential monitoring of SII.

This study has several limitations. First, as an observational study, residual confounding may persist despite adjustments for significant confounders, potentially affecting causal inference. Second, the single-center design limits the generalizability of our findings to broader populations. Third, the retrospective and acute-care nature of the study prevented verification of participants' fasting status before blood sampling, possibly introducing bias in glucose and derived indices like TyG. Fourth, extrapolating results to populations with diverse regional or ethnic characteristics requires caution. Additionally, while the derived neutrophil-to-lymphocyte ratio (dNLR) was assessed among inflammatory indices, its approximation of lymphocyte count may be influenced by other cell counts; however, as a non-primary marker, this does not significantly impact our main findings. Furthermore, incomplete data collection during 2013–2018 led to the unavailability of key biomarkers like high-sensitivity C-reactive protein (hs-CRP), and inconsistent clinical documentation resulted in the absence of detailed angiographic parameters (e.g., TIMI flow grades, Gensini score). These limitations are noted in the manuscript, and we recommend future studies include comprehensive assessments of inflammatory markers and angiographic metrics. Lastly, advances in interventional cardiology during the study period (June 2013 to February 2018) may have influenced patient outcomes.

## 5 Conclusion

This study observed distinct mechanisms of glucose-lipid metabolism and systemic inflammation in the mortality risk of ACS patients: the TyG index independently increased risk through direct glucose toxicity. In contrast, lipid metabolism associated with the AIP exhibited both direct lipotoxic effects and SII-mediated compensatory anti-inflammatory effects. The relationship between SII and mortality demonstrated a U-shaped nonlinear pattern, indicating that dysregulation of inflammatory balance results in bidirectional harm. Age ≥75 years, STEMI diagnosis, and elevated TC were identified as independent risk factors for mortality in ACS patients. Elevated HDL-C was an independent protective factor for mortality in ACS patients. The study emphasizes the necessity of dynamic monitoring of the metabolic-inflammator*y* axis and dual-pathway targeted interventions during acute cardiac events.

## Data Availability

The datasets used and/or analysed during the current study are available from the corresponding author on reasonable request.

## Glossary

ACME: average causal mediation effect
ACS: acute coronary syndrome
ADE: average direct effect
AIC: akaike information criterion
AIP: atherogenic index of plasma
ALB: albumin
ALT: alanine aminotransferase
AMI: acute myocardial infarction
apoA1: apolipoprotein A1
apoB: apolipoprotein B
ASCVD: atherosclerotic cardiovascular disease
AST: aspartate aminotransferase
BUN: blood urea nitrogen
CBC: complete blood count
ChE: cholinesterase
CI: confidence interval
Crea: creatinine
CVD: cardiovascular disease
D-Dimer: D-Dimer fragment
dNLR: derived neutrophil-to-lymphocyte ratio
FDP: fibrin degradation products
GLU: glucose
Hct: hematocrit
HDL-C: high-density lipoprotein cholesterol
HOMA-IR: Homeostasis model assessment of insulin resistance
HR: hazard ratio
Hs-CRP: high-sensitivity C-reactive protein
LASSO: least absolute shrinkage and selection operator
LDL-C: low-density lipoprotein cholesterol
LPa: lipoprotein a
MACE: major adverse cardiovascular events
MCHC: mean corpuscular hemoglobin concentration
MLR: monocyte-to-lymphocyte ratio
NLR: neutrophil-to-lymphocyte ratio
NMLR: neutrophil-to-monocyte-to-lymphocyte ratio
NSTEMI: non-ST-segment elevation myocardial infarction
n-3 PUFAs: n-3 polyunsaturated fatty acids
OR: odds ratio
PCI: percutaneous coronary intervention
PPAR-γ: peroxisome proliferator-activated receptor gamma
RCS: restricted cubic splines
RDW-CV: red cell distribution width-coefficient of variation
ROS: reactive oxygen species
S1P: sphingosine-1-phosphate
SII: systemic immune-inflammation index
SIRI: systemic inflammation response index
STEMI: ST-segment elevation myocardial infarction
TC: total cholesterol
TG: triglycerides
TGRL: triglyceride-rich lipoprotein
TyG: triglyceride-glucose index
UA: unstable angina
WBC: white blood cell
