# Supplementary material for: The association between triglyceride-glucose index, atherogenic index of plasma, systemic immune-inflammation index, and mortality in patients with acute coronary syndrome: the direct effects of glucose-lipid metabolism and U-shaped immune modulation in mortality risk
Source: Front Cardiovasc Med. 2025 Jul 25;12:1604284. doi: 10.3389/fcvm.2025.1604284 (PMC12331594; doi:10.3389/fcvm.2025.1604284)
Supplement: Supplementary file 1 [file Table1.docx]

**Supplementary Table 1.** Clinical data of patients with ACS stratified by mortality status (N=3861) (*p<0.05, **p<0.01,***p<0.001)

| Variables | Death  N=206 | Alive  N=3655 | p value |
| --- | --- | --- | --- |
| Pulse | 86.28±21.77 | 77.13±15.03 | *<0.001**** |
| Temperature | 36.59±0.53 | 36.40±0.40 | *<0.001**** |
| Respiratory_rate | 19.60±4.67 | 18.51±5.27 | *0.001*** |
| Hospitalization_days | 8(2,17) | 7(5,11) | *0.666* |
| ALT(U/L) | 31(14.75 , 60.50 ) | 24(15 , 40) | *0.001*** |
| LDH(U/L) | 300.5(209 , 621) | 217(168 , 360) | *<0.001**** |
| LDLC(mmol/L) | 2.36±0.87 | 2.61±0.89 | *<0.001**** |
| AST(U/L) | 44.5(23 , 130.75) | 28(19 , 70) | *<0.001**** |
| BUN(mmol/L) | 12.77±10.42 | 6.99±4.83 | *<0.001**** |
| UA(μmol/L) | 410.08±177.98 | 347.72±117.61 | *<0.001**** |
| TC(mmol/L) | 4.03±1.25 | 4.29±1.18 | *0.003*** |
| TBiL(μmol/L) | 12.45(8.28 , 18.23) | 11.60(8.50 , 15.80) | *0.043** |
| TBA(μmol/L) | 4.05(2.20 , 7.35) | 3.63(2.12 , 5.84 ) | *0.015** |
| TP(g/L) | 60.59±8.39 | 63.68±6.38 | *<0.001**** |
| TG(mmol/L) | 1.63±1.44 | 1.72±1.35 | *0.39* |
| ALB(g/L) | 33.08±6.22 | 37.81±4.91 | *<0.001**** |
| DBiL(μmol/L) | 3.35(1.70 , 5.80) | 3.33(2.27 , 4.70) | *0.576* |
| ALP(U/L) | 85.01±66.44 | 75.14±38.04 | *0.036** |
| Crea(μmol/L) | 126(89.75 , 202.25) | 82(68 ,103) | *<0.001**** |
| CK(U/L) | 199.5(68 , 900) | 118(68 , 426) | *0.004*** |
| ChE(U/L) | 5424.79±2164.53 | 7433.63±2118.64 | *<0.001**** |
| LPa(mg/L) | 367.14±312.49 | 334.92±286.32 | *0.118* |
| Glucose(mmol/L) | 10.44±6.37 | 8.01±3.92 | *<0.001**** |
| apoB(g/L) | 0.72±0.23 | 0.78±0.22 | *<0.001**** |
| apoA1(g/L) | 0.88±0.28 | 1.00±0.26 | *<0.001**** |
| HDLC(mmol/L) | 1.01±0.28 | 1.09±0.27 | *<0.001**** |
| Neut(10^9/L) | 9.53±6.22 | 6.26±3.57 | *<0.001**** |
| Mono(10^9/L) | 0.52(0.35 , 0.73) | 0.42(0.31 , 0.56) | *<0.001**** |
| Baso(10^9/L) | 0.01(0.01 , 0.02) | 0.02(0.01 , 0.03) | *<0.001**** |
| Eos(10^9/L) | 0.01(0 , 0.07) | 0.07(0.03 , 0.15) | *<0.001**** |
| P_LCR％ | 32.51±10.77 | 30.56±9.28 | *0.011** |
| MCV(ft) | 90.07±6.89 | 89.96±5.21 | *0.81* |
| MCH(pg) | 29.69±2.47 | 30.18±2.09 | *0.005*** |
| MCHC(g/L) | 329.33±14.08 | 335.53±12.14 | *<0.001**** |
| MPV(fL) | 10.89±1.45 | 10.63±1.34 | *0.008*** |
| WBC(10^9/L) | 11.51±6.72 | 8.35±3.73 | *<0.001**** |
| Lymph(10^9/L) | 1.22±0.69 | 1.48±0.66 | *<0.001**** |
| RDWCV％ | 14.2±1.96 | 13.30±1.16 | *<0.001**** |
| RDWSD(fL) | 46.07±6.72 | 43.50±4.03 | *<0.001**** |
| Hct% | 18.95(0.35 , 36.03) | 32.13(0.41 , 40.72) | *<0.001**** |
| PLT(10^9/L) | 184.58±81.35 | 198.54±64.59 | *0.016** |
| PDW(fL) | 15.02±2.49 | 14.68±2.32 | *0.04** |
| PCT% | 0.20±0.08 | 0.21±0.06 | *0.049** |
| Hb(g/L) | 115.26±26.25 | 132.21±21.31 | *<0.001**** |
| PT(s) | 14.49±10.36 | 12.25±6.27 | *0.002*** |
| D_Dimer(μg/L) | 672.51(270.50 , 1709.25) | 160(78 , 351) | *<0.001**** |
| PT_INR(mg/L) | 1.31±0.79 | 1.14±0.62 | *0.003*** |
| FDP | 5.41(2.79 , 12.87) | 1.84(1.03 , 3.16) | *<0.001**** |
| Fbg(g/L) | 3.86±1.01 | 3.81±0.86 | *0.478* |
| NLR | 8.60(3.83 , 14.00) | 3.73(2.43 , 6.37) | *<0.001**** |
| dNLR | 0.93±0.20 | 0.90±0.11 | *0.024** |
| MLR | 0.61±0.46 | 0.38±0.34 | *<0.001**** |
| NMLR | 8.98(4.17 , 14.88) | 4.05(2.68 , 6.83) | *<0.001**** |
| SIRI | 3.85(1.76 , 8.27) | 1.56(0.89 , 3.00) | *<0.001**** |
| SII | 1135.86(593.23 , 2784.61) | 716.62(441.19 , 1287.06) | *<0.001**** |
| TyG | 9.20±0.82 | 9.04±0.72 | *0.007*** |
| AIP | 0.51±0.26 | 0.49±0.25 | *0.367* |
| Pulse | 86.28±21.77 | 77.13±15.03 | *<0.001**** |
| Temperature | 36.59±0.53 | 36.40±0.40 | *<0.001**** |
| Respiratory_rate | 19.60±4.67 | 18.51±5.27 | *0.001*** |
| Hospitalization_days | 8(2,17) | 7(5,11) | *0.666* |
| ALT(U/L) | 31(14.75 , 60.50 ) | 24(15 , 40) | *0.001*** |
| LDH(U/L) | 300.5(209 , 621) | 217(168 , 360) | *<0.001**** |
| LDLC(mmol/L) | 2.36±0.87 | 2.61±0.89 | *<0.001**** |

Abbreviations: Pulse: Pulse rate, Temperature: Body temperature, Respiratory_rate: Respiratory rate, Hospitalization_days: Duration of hospitalization, ALT: Alanine aminotransferase, LDH: Lactate dehydrogenase, LDLC: Low-density lipoprotein cholesterol, AST: Aspartate aminotransferase, BUN: Blood urea nitrogen, UA: Uric acid, TC: Total cholesterol, TBiL: Total bilirubin, TBA: Total bile acid, TP: Total protein, TG: Triglyceride, ALB: Albumin, DBiL: Direct bilirubin, ALP: Alkaline phosphatase, Crea: Creatinine, CK: Creatine kinase, ChE: Cholinesterase, LPa: Lipoprotein(a), Glucose: Blood glucose, apoB: Apolipoprotein B, apoA1: Apolipoprotein A1, HDLC: High-density lipoprotein cholesterol, Neut: Neutrophils, Mono: Monocytes, Baso: Basophils, Eos: Eosinophils, P_LCR: Platelet large cell ratio, MCV: Mean corpuscular volume, MCH: Mean corpuscular hemoglobin, MCHC: Mean corpuscular hemoglobin concentration, MPV: Mean platelet volume, WBC: White blood cells, Lymph: Lymphocytes, RDWCV: Red cell distribution width coefficient of variation, RDWSD: Red cell distribution width standard deviation, Hct: Hematocrit, PLT: Platelets, PDW: Platelet distribution width, PCT: Plateletcrit, Hb: Hemoglobin, PT: Prothrombin time, D_Dimer: D-dimer, PT_INR: Prothrombin time international normalized ratio, FDP: Fibrin degradation products, Fbg: Fibrinogen, NLR: Neutrophil-to-lymphocyte ratio, dNLR: Derived neutrophil-to-lymphocyte ratio, MLR: Monocyte-to-lymphocyte ratio, NMLR: Neutrophil-monocyte-to-lymphocyte ratio, SIRI: Systemic immune-inflammation index, SII: Systemic immune-inflammation index, TyG: Triglyceride-glucose index, AIP: Atherogenic index of plasma.
