# Supplementary material for: The association between triglyceride-glucose index, atherogenic index of plasma, systemic immune-inflammation index, and mortality in patients with acute coronary syndrome: the direct effects of glucose-lipid metabolism and U-shaped immune modulation in mortality risk
Source: Front Cardiovasc Med. 2025 Jul 25;12:1604284. doi: 10.3389/fcvm.2025.1604284 (PMC12331594; doi:10.3389/fcvm.2025.1604284)
Supplement: Supplementary file 2 [file Table3.docx]

**Supplementary Table 3.** Clinical data of patients with AMI stratified by mortality status (N=2398) (*p<0.05, **p<0.01,***p<0.001)

| Variables | Death  N=182 | Alive  N=2261 | p value |
| --- | --- | --- | --- |
| Pulse | 86.31±21.78 | 79.16±15.98 | *<0.001**** |
| Temperature | 36.58±0.55 | 36.40±0.41 | *<0.001**** |
| Respiratory_rate | 19.53±4.70 | 18.76±5.64 | *0.037** |
| Hospitalization_days | 8(2 ,18) | 7(5 ,11) | *0.515* |
| ALT(U/L) | 32(16 , 62.52) | 29(18 ,47) | *0.111* |
| LDH(U/L) | 320.5(210 , 692) | 290(200 , 520.75) | *0.036** |
| LDLC(mmol/L) | 2.38±0.85 | 2.74±0.89 | *<0.001**** |
| AST(U/L) | 51.51(25.00 , 154.53) | 45(24 , 117) | *0.209* |
| BUN(mmol/L) | 12.96±10.89 | 7.17±5.16 | *<0.001**** |
| UA(μmol/L) | 416.58±182.11 | 352.27±120.66 | *<0.001**** |
| TC(mmol/L) | 4.04±1.21 | 4.41±1.17 | *<0.001**** |
| TBiL(μmol/L) | 16.47±16.41 | 13.94±12.49 | *0.044** |
| TBA(μmol/L) | 4.10(2.33 , 7.35) | 3.27(1.90 , 5.23) | *<0.001**** |
| TP(g/L) | 60.79±8.37 | 62.73±6.28 | *0.003*** |
| TG(mmol/L) | 1.63±1.49 | 1.75±1.45 | *0.273* |
| ALB(g/L) | 33.19±6.12 | 37.27±4.93 | *<0.001**** |
| DBiL(μmol/L) | 3.38(1.58 , 5.63) | 3.32(2.25 , 4.76) | *0.947* |
| ALP(U/L) | 80.95±45.24 | 76.06±35.22 | *0.156* |
| Crea(μmol/L) | 127(93 , 205) | 84(68 , 107) | *<0.001**** |
| CK(U/L) | 266(85 , 1193.75) | 241(93 , 1009.52) | *0.962* |
| ChE(U/L) | 5491.27±2181.98 | 7366.45±2165.39 | *<0.001**** |
| LPa(mg/L) | 372.91±312.24 | 337.43±287.34 | *0.112* |
| Glucose(mmol/L) | 10.68±6.51 | 8.26±4.04 | *<0.001**** |
| apoB(g/L) | 0.73±0.23 | 0.81±0.23 | *<0.001**** |
| apoA1(g/L) | 0.88±0.27 | 0.99±0.26 | *<0.001**** |
| HDLC(mmol/L) | 1.00±0.27 | 1.08±0.26 | *<0.001**** |
| Neut(10^9/L) | 9.90±6.19 | 7.20±3.91 | *<0.001**** |
| Mono(10^9/L) | 0.53(0.37 , 0.73) | 0.45(0.33 , 0.61) | *<0.001**** |
| Baso(10^9/L) | 0.01(0.01 , 0.02) | 0.02(0.01 , 0.03) | *<0.001**** |
| Eos(10^9/L) | 0.01(0 , 0.06) | 0.06(0.02 , 0.13) | *<0.001**** |
| P_LCR％ | 32.74±10.83 | 30.76±9.39 | *0.017** |
| MCV(ft) | 90.22±7.06 | 89.86±5.26 | *0.509* |
| MCH(pg) | 29.72±2.54 | 30.17±2.09 | *0.02** |
| MCHC(g/L) | 329.29±14.35 | 335.80±12.05 | *<0.001**** |
| MPV(fL) | 10.94±1.45 | 10.65±1.38 | *0.007*** |
| WBC(10^9/L) | 11.78±6.38 | 9.27±4.09 | *<0.001**** |
| Lymph(10^9/L) | 1.24±0.70 | 1.45±0.69 | *<0.001**** |
| RDWCV％ | 14.17±2.02 | 13.30±1.15 | *<0.001**** |
| RDWSD(fL) | 46.03±6.96 | 43.52±4.01 | *<0.001**** |
| Hct% | 16.22(0.35 , 35.53) | 28.84(0.43 , 39.80) | *<0.001**** |
| PLT(10^9/L) | 187.64±80.81 | 202.00±66.25 | *0.021** |
| PDW(fL) | 15.00±2.55 | 14.78±2.33 | *0.222* |
| PCT% | 0.20±0.08 | 0.21±0.07 | *0.069* |
| Hb(g/L) | 115.67±26.56 | 131.30±21.97 | *<0.001**** |
| PT(s) | 14.63±10.77 | 12.73±7.48 | *0.02** |
| D_Dimer(μg/L) | 650.5(268.25 , 1465) | 168(84 , 389) | *<0.001**** |
| PT_INR(mg/L) | 1.32±0.83 | 1.19±0.76 | *0.04** |
| FDP | 5.39(2.93 , 12.87) | 1.94(1.09 , 3.47) | *<0.001**** |
| Fbg(g/L) | 3.89±1.02 | 3.89±0.87 | *0.952* |
| SII | 1190.92(658.64,2842.86) | 869.51(533.45,1566.01) | *<0.001**** |
| TyG | 9.22±0.81 | 9.09±0.73 | *0.017** |
| AIP | 0.51±0.26 | 0.50±0.26 | *0.689* |

Abbreviations: Pulse: Pulse rate, Temperature: Body temperature, Respiratory_rate: Respiratory rate, Hospitalization_days: Duration of hospitalization, ALT: Alanine aminotransferase, LDH: Lactate dehydrogenase, LDLC: Low-density lipoprotein cholesterol, AST: Aspartate aminotransferase, BUN: Blood urea nitrogen, UA: Uric acid, TC: Total cholesterol, TBiL: Total bilirubin, TBA: Total bile acid, TP: Total protein, TG: Triglyceride, ALB: Albumin, DBiL: Direct bilirubin, ALP: Alkaline phosphatase, Crea: Creatinine, CK: Creatine kinase, ChE: Cholinesterase, LPa: Lipoprotein(a), Glucose: Blood glucose, apoB: Apolipoprotein B, apoA1: Apolipoprotein A1, HDLC: High-density lipoprotein cholesterol, Neut: Neutrophils, Mono: Monocytes, Baso: Basophils, Eos: Eosinophils, P_LCR: Platelet large cell ratio, MCV: Mean corpuscular volume, MCH: Mean corpuscular hemoglobin, MCHC: Mean corpuscular hemoglobin concentration, MPV: Mean platelet volume, WBC: White blood cells, Lymph: Lymphocytes, RDWCV: Red cell distribution width coefficient of variation, RDWSD: Red cell distribution width standard deviation, Hct: Hematocrit, PLT: Platelets, PDW: Platelet distribution width, PCT: Plateletcrit, Hb: Hemoglobin, PT: Prothrombin time, D_Dimer: D-dimer, PT_INR: Prothrombin time international normalized ratio, FDP: Fibrin degradation products, Fbg: Fibrinogen, SII: Systemic immune-inflammation index, TyG: Triglyceride-glucose index, AIP: Atherogenic index of plasma.
