# Supplementary material for: The association between triglyceride-glucose index, atherogenic index of plasma, systemic immune-inflammation index, and mortality in patients with acute coronary syndrome: the direct effects of glucose-lipid metabolism and U-shaped immune modulation in mortality risk
Source: Front Cardiovasc Med. 2025 Jul 25;12:1604284. doi: 10.3389/fcvm.2025.1604284 (PMC12331594; doi:10.3389/fcvm.2025.1604284)
Supplement: Supplementary file 3 [file Table2.docx]

**Supplementary Table 2.** Variables Selected by LASSO Regression

| Variable Name | Unit | Variable Type |
| --- | --- | --- |
| Age | years | Continuous |
| Sex | N/A (Male/Female) | Categorical |
| Diabetes Mellitus | N/A (Yes/No) | Categorical |
| Smoking Status | N/A (Ever/Never) | Categorical |
| Heart Rate | beats per minute (bpm) | Continuous |
| Albumin (ALB) | g/L | Continuous |
| Blood Urea Nitrogen (BUN) | mmol/L | Continuous |
| Cholinesterase (ChE) | U/L | Continuous |
| D-Dimer Fragment (D-Dimer) | μg/L | Continuous |
