# Supplementary material for: The association between triglyceride-glucose index, atherogenic index of plasma, systemic immune-inflammation index, and mortality in patients with acute coronary syndrome: the direct effects of glucose-lipid metabolism and U-shaped immune modulation in mortality risk
Source: Front Cardiovasc Med. 2025 Jul 25;12:1604284. doi: 10.3389/fcvm.2025.1604284 (PMC12331594; doi:10.3389/fcvm.2025.1604284)
Supplement: Supplementary file 4 [file Image1.pdf]

Supplementary Figure 1. Associations between SII, TyG and AIP and mortality in AMI patients

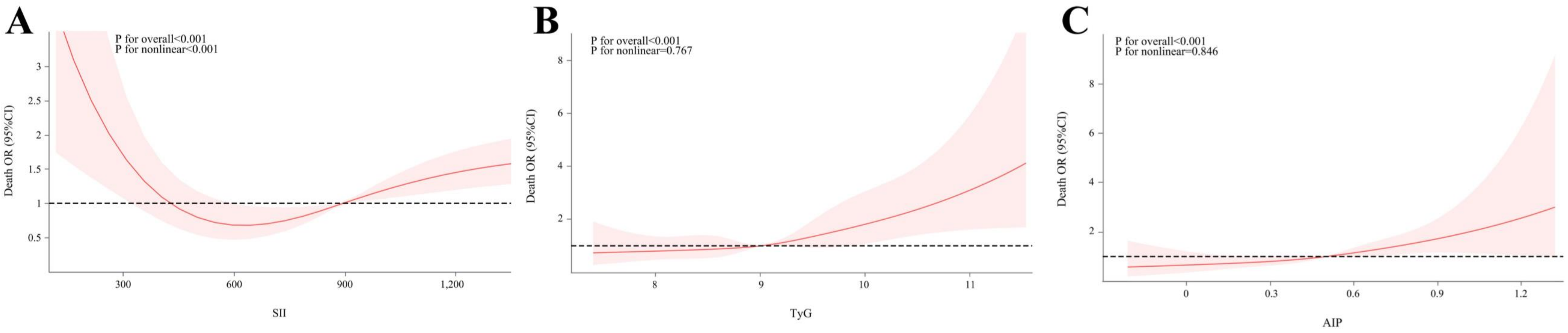

Figure caption： The relationships between SII, TyG, and AIP and mortality in the AMI patient population were analyzed by assessing overall significance and nonlinear significance. P values were calculated for overall effect and nonlinearity. Solid lines in the corresponding graphs indicate estimated relationships and shaded areas indicate 95% confidence intervals.
